# Intensity Modulated Radiotherapy (IMRT) + Carbon Ion Boost for Adenoid Cystic Carcinoma of the Minor Salivary Glands in the Oral Cavity

**DOI:** 10.3390/cancers10120488

**Published:** 2018-12-04

**Authors:** Kristin Lang, Melissa Baur, Sati Akbaba, Thomas Held, Steffen Kargus, Nina Bougatf, Denise Bernhardt, Kolja Freier, Peter K. Plinkert, Stefan Rieken, Jürgen Debus, Sebastian Adeberg

**Affiliations:** 1 Department of Radiation Oncology, Heidelberg University Hospital, Im Neuenheimer Feld 400, 69120 Heidelberg, Germany; kristin.lang@med.uni-heidelberg.de (K.L.); baur@stud.uni-heidelberg.de (M.B.); sati.akbaba@med.uni-heidelberg.de (S.A.); thomas.held@med.uni-heidelberg.de (T.H.); nina.bougatf@med.uni-heidelberg.de (N.B.); denise.bernhardt@med.uni-heidelberg.de (D.B.); stefan.rieken@med.uni-heidelberg.de (S.R.); juergen.debus@med.uni-heidelberg.de (J.D.); 2 Heidelberg Ion-Beam Therapy Center, 69120 Heidelberg, Germany; 3 Heidelberg Institute of Radiation Oncology, 69120 Heidelberg, Germany; 4 Department of Oral and Maxillofacial Surgery, University Hospital Heidelberg, Im Neuenheimer Feld 400, 69120 Heidelberg, Germany; steffen.kargus@med.uni-heidelberg.de (S.K.); kolja.freier@med.uni-heidelberg.de (K.F.); 5 Department of Otorhinolaryngology, Head and Neck Surgery, University Hospital Heidelberg, Im Neuenheimer Feld 400, 69120 Heidelberg, Germany; peter.plinkert@med.uni-heidelberg.de

**Keywords:** intensity modulated radiotherapy, carbon ions, perineural invasion, ACC, postoperative

## Abstract

Background: Adenoid cystic carcinoma (ACC) are more common in the minor salivary glands (MiSGs) than the major salivary glands, and are characterized by slow tumor progression and frequently local recurrence. The main treatment option is surgery followed by combined radiotherapy. Methods: A retrospective analysis contained 67 patients with ACC of MiSGs in the oral cavity who underwent surgery followed by radiotherapy. The median cumulative IMRT dose was 50 Gy followed by 24 Gy for carbon ion (C12) boost. Median follow-up was 40 months. Results: Median 5-years overall survival (OS), progression-free survival (PFS) and local disease-free survival (LDFS) rates were 85.5%, 57.4% and 74.9%. Median time until progression was detected was 32 months (range: 2–205 months). Early grade ≥3 mucositis, dermatitis, and dysphagia were detected in 52.2%, 7.5% and 11.9% respectively. Besides common toxicities, two patients (3.0%) developed grade 3 toxicities with osteoradionecrosis of the jaw after 18 and 66 months. Higher-grade late toxicity (CTCAE grade 4) was not detected. No treatment-related death was detected. Conclusions: Our results demonstrate that postoperative combined radiotherapy with IMRT plus C12 boost seems to be a feasible and effective treatment method in ACC of MiSGs in the oral cavity, with good control and survival rates and adequate toxicity.

## 1. Introduction

Adenoid cystic carcinomas (ACCs) of the head and neck are located in the major salivary glands (MaSgs) and in the minor salivary glands (MiSGs). MiSGs are more often involved than MaSgs. ACCs of MiSGs are represented with 2–4% of all head and neck cancers and 50% of ACCs arise in the minor salivary glands [1,2,3]. The most frequent involved site in the oral cavity is the palate [1,4]. In general, ACCs are characterized by slow tumor progression associated with pain due to the predisposition of perineural invasion. Mean patient age is the middle and older years [5], and ACCs have a tendency for local recurrence and distant metastases. So far, long-term prognoses are poor [6]. Distant metastases occur in 40–60% of patients, especially in late-stage disease [3,7,8,9]. The standard treatment strategy for ACCs is radical resection followed by adjuvant high-dose radiotherapy, especially in advanced disease (T3–4) and positive resection margin (R1–2) [10,11]. Some studies report better survival in patients who undergo RT with positive lymph nodes compared with lymph node negative patients [12]. Over the last years, combined radiotherapy (RT) with photons and boost RT with carbon ions (C12) has been investigated and has shown favorable outcomes compared with photons alone for ACCs [13] (Table 1). The C12 therapy is seen as a method of dose escalation in the tumor and/or the tumor bed. As a standard, patients receive a combined treatment regimen consisting of an up-front carbon ion boost followed by standard IMRT techniques [12]. To achieve long-term local control, high total doses more than 60 Gy are necessary, but application is critical in view of the proximity to critical structures in oral cavity [14,15]. This study summarizes our institution’s experience with combined postoperative RT in ACCs of the minor salivary glands in the oral cavity to evaluate survival and toxicity.

## 2. Results

### 2.1. Treatment Outcome

After a median follow-up of 40 months (range: 1–200), 58 patients (86.6%) were still alive. Nine patients died: three due to disease progression, the other six patients because of pulmonary infection and cardiac disease. Tumor progression could be observed in 19 patients (28.4%), 9 patients (13.4%) had local recurrence, and 9 patients (13.4%) developed distant metastases, while 1 patient (1.5%) developed local tumor progression as well as distant metastases. The most common location of distant metastasis was the lungs (85.7%), followed by the bone (14.3%), which occurred in a median time span of 24 months after RT (range 1–73 months).

**Figure 1:**
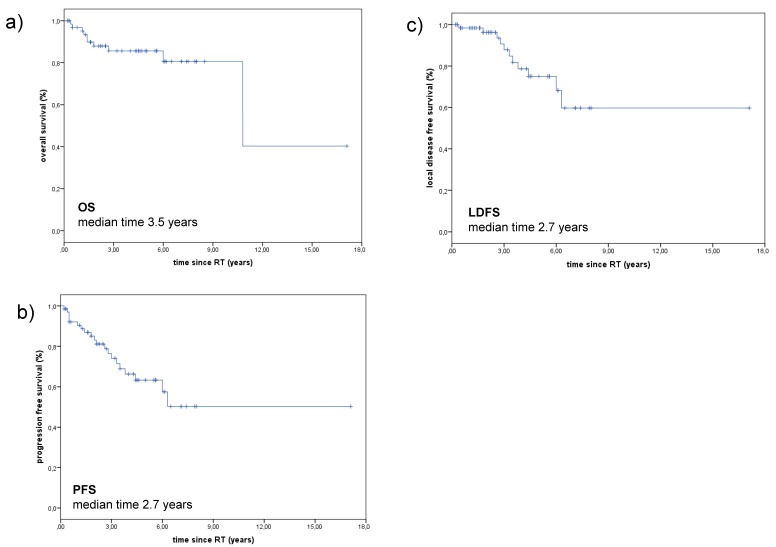
OS, PFS and LDFS of patients with ACCs of the MiSGs (**a**) OS of patients with ACC of the MiSGs in oral cavity after RT. The 3-year Kaplan-Meier estimates for OS was 85.6% and the median OS was 3.5 years. (**b**) PFS of patients with ACC of the minor salivary glands in oral cavity. Median PFS was 2.7 years, the 3-year Kaplan-Meier estimates for PFS was 74.0%. (**c**) LDFS of patients with ACC of the minor salivary glands in oral cavity. Median LDFS was 2.7 years, the 3-year Kaplan-Meier estimates for LDFS was 87.8%. Abbreviations: overall survival (OS), progression-free survival (PFS), local disease-free survival (LDFS), adenoid cystic carcinoma (ACC), minor salivary glands (MiSGs).

The median PFS and LDFS were 2.7 years (range: 0.2–17.1 years) and median OS since RT was 3.5 years (range: 0.2–17.1 years). The 3-year Kaplan-Meier estimates for OS, PFS, and LDFS were 85.5%, 74.0%, and 87.8%, respectively, and 10-year OS, PFS, and LDFS rates were 63.1%, 50.2%, and 59.6% respectively (Figure 1).

The median time until progression of the disease was 24 months (range: 1–73), median time for local recurrence was 41 months (range: 1–73), and median time for distant metastases was 64 months (range: 31–127).

### 2.2. Prognostic Factors

**Figure 2:**
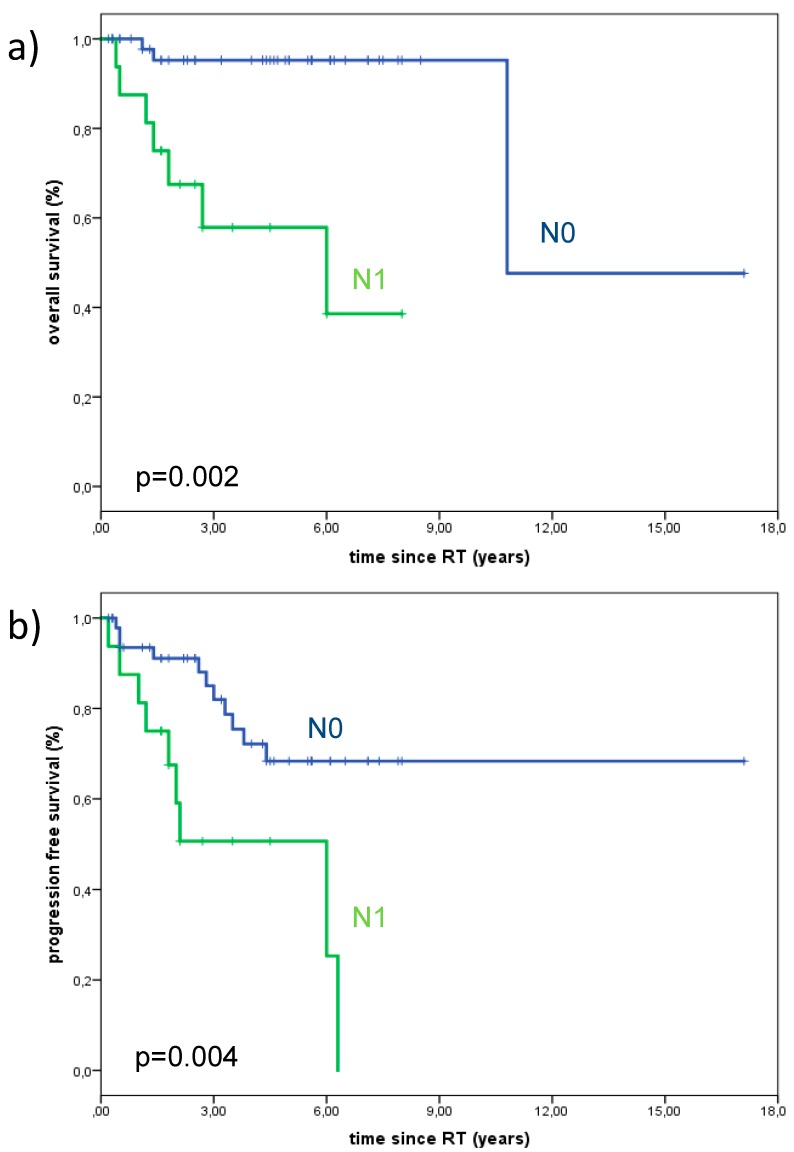
Overall survival and progression-free survival of 67 patients treated with curative intent for minor salivary gland adenoid cystic carcinoma with significant better survival and less progressive disease in patients with lymph node negative status (*p* = 0.002 and 0.004). a) The 3-year Kaplan-Meier estimates for OS in lymph node negative vs. lymph node positive patients were 95.2% vs. 57.9%, and b) the 3-year Kaplan-Meier estimates for PFS in lymph node negative vs. lymph node positive patients were 82.0% vs. 50.6%. Abbreviations: overall survival (OS), progression-free survival (PFS).

**Figure 3:**
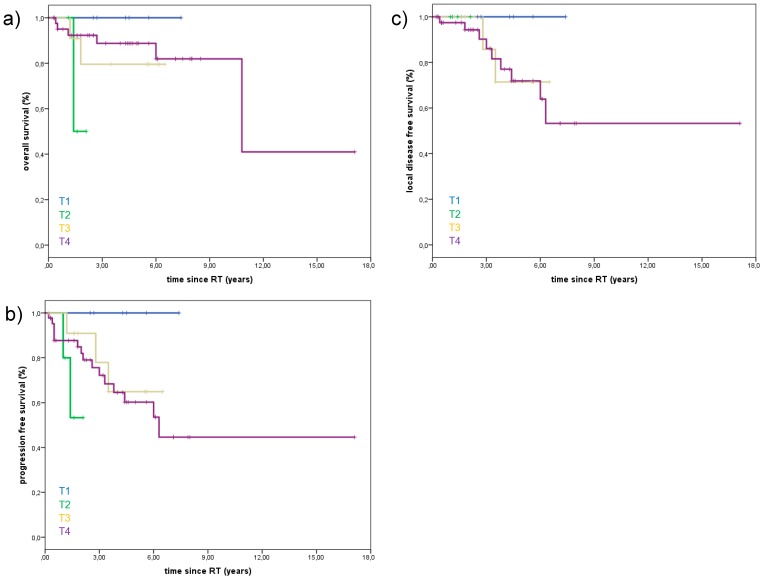
Overall survival (OS), progression-free survival (PFS), and local disease-free survival (LDFS) of 67 patients treated with curative intent for minor salivary gland adenoid cystic carcinoma with not significant difference between T-stages (T1–4). (**a**) Death during follow up, T1: o/6 patients (0%), T2: 2/5 patients (40%), T3:2/12 patients (16.7%), T4: 6/44 patients (13.6%). (**b**) Progression of disease during follow up (local and distant), T1: o/6 patients (0%), T2: 2/5 patients (40%), T3: 3/12 patients (25%), T4: 15/44 patients (34.1%). (**c**) Local recurrence during follow up, T1: o/6 patients (0%), T2: 0/5 patients (0%), T3: 2/12 patients 16.7%), T4: 9/44 patients (20.5%). Abbreviations: overall survival (OS), progression-free survival (PFS), local disease-free survival (LDFS).

Univariate and multivariate analyses were carried out to explore potential prognosticators for local control and OS among subgroups. Lymph node positive patients were associated with significantly poorer OS and PFS. The 3-year Kaplan-Meier estimates for OS in lymph node negative vs. lymph node positive patients were 95.2% vs. 57.9% (*p* = 0.002), and the 3-year Kaplan-Meier estimates for PFS in lymph node negative vs. lymph node positive patients were 82.0% vs. 50.6%. (*p* = 0.004) (Figure 2). However, neural invasion was not significantly associated with the rate of locoregional recurrences. The presence of distant metastases during follow-up was associated with poorer OS. OS, PFS, and LDFS were not significantly dependent upon T-stage. During follow up, no patient with T1-stage died, while 13.6% with T4-stage died. OS, PFS, and LDFS for the different T-stages are summarized in Figure 3. PFS, OS, and LDFS didn’t not show any significant difference regarding resection status. Young age, female gender, total doses ≤ 72Gy, and CTV volume (ccm) were not predictors for improved LC, PFS, or OS.

### 2.3. Treatment Toxicity

**Figure 4:**
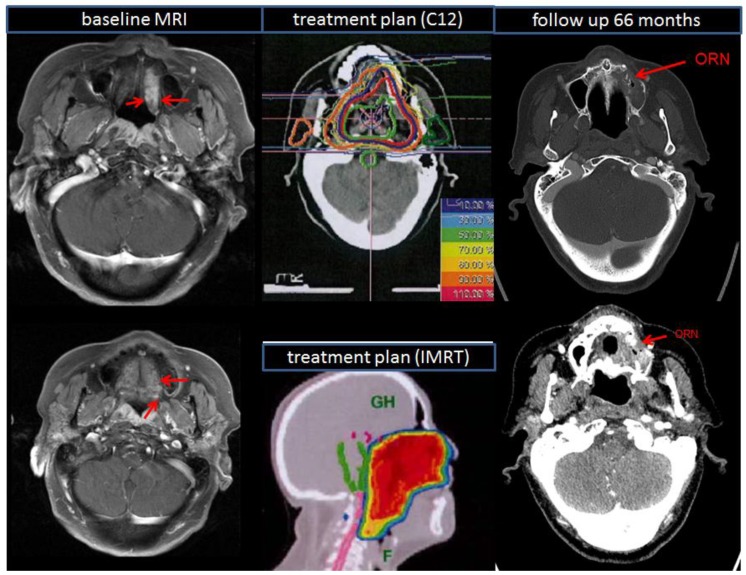
Radiation-induced osteoradionecrosis of the upper jaw: baseline MRI scan (left side) and follow-up CT scan (right side) of a patient who undergone surgery of adenoid cystic carcinoma of hard palate and additive bimodal radiotherapy. 66 months after RT, there was increased soft tissue as well as erosion of the posterior wall of the left side of maxilla. In comparison with the initially-treated radiation plan, the lesion occurred in an irradiated region of the hard palate at the edge of the 95%-isodose (middle up: carbon ion isodose plan alone, middle down: IMRT isodose plan alone). Abbreviations: radiotherapy (RT), computed tomography (CT), magnetic resonance imaging (MRI), intensity modulated radiotherapy (IMRT), carbon ions (C12).

Most common acute RT-related complications were dermatitis (56.7%), oral mucositis (31.3%), dysphagia (58.2%), trismus (23.9%), xerostomia (64.2%), and loss of taste (74.6%), summarized in Table 2. Middle-ear effusions (19.4%) with hearing impairment were not uncommon. There were no treatment-related deaths. Late RT-related complications occurred in the form of xerostomia (49.3%), dermatitis (16.4%), and trismus (29.9%). Also, 13% of the patients suffered from persisting moderate fatigue 12 months after RT. Higher-grade late toxicity (CTCAE grade 4) was not detected. However, we observed in the course of the follow-ups two cases (3.0%) of osteoradionecrosis (ORN) of the lower jaw (grade 3) (Figure 4): one patient developed an ORN after 18 month, the other after 66 months. In both cases, ORN presented with wide pain, especially during eating. They underwent another biopsy and debridement of the left side of maxilla. Intraoperatively, necrotic bone in the posterior wall and lateral buttress of the maxilla were debrided along with polypoid, friable granulation tissue, which was sent for pathology and culture.

## 3. Discussion

This study demonstrates that combined RT with photons and carbon ions of ACCs of the MiSGs leads to good local control rates, and that lymph node status is a prognostic factor for PFS and OS. Target volume measures did not influence survival rates. The acute and chronic toxicity was low; only two patients developed ORN.

The median follow-up of only 40 months seems to be relatively short, but this is due to the original goal of this work to investigate modern radiation techniques. Over recent years, not least from our institution, several studies have reported on the outcome of ACCs in MiSGs in different locations. The present study shows solely the results from patients with ACCs of MiSGs in the oral cavity which are more often involved than the MaSGs. A multimodal approach of surgery and combined RT (IMRT plus carbon ion boost) is thought to be the most effective. Radiotherapy aims to reduce the risk of local recurrence [16,17,18,19,20,21]. All patients in the presented study were regularly treated with IMRT plus carbon ion (C12) boost based on CT and MRI scans for planning and same target volume definition principles after radical surgery. After treatment, the patients underwent regular follow up CT and MRI scans at our institution.

Overall, the results in the present study are comparable to those reported in previous studies. The adjuvant treatment results in good local control, with 5 and 10-year survival rates of 85.5% and 63.1% respectively. Previous studies reported about OS between 60–90% at 5 years and 40–60% at 10 years. These values are also comparable with studies involving our institution’s earlier research. Jensen et al. analyzed similar results as ours towards 5-year OS, PFS, and LDFS [1,8,15,22,23].

In previous studies, ACC has shown female predominance which was also detected in the present results (59.7%) [3,24]. In the current study, gender was not associated with better OS (*p* = 0.437), which is in contrast to the reported literature. This is possible because all reported studies include series of all head and neck ACCs as well as ACCs in the neck and pharynx [18]. Our presented patient cohort showed a median age of 58 years, which is the typical age for first diagnosis of ACCs of MiSGs in the oral cavity [3,24,25].

The observed OS rate of 100% in patients with T1 stage seems to be very promising, but has to be put into perspective of the relatively short follow-up interval, as well as the insubstantial number of patients with T1 and T2 stages. In contrast to our study where T-stages did not significantly affect OS, LDFS, and PFS, DeAngelis et al. showed in a univariate analysis a significant prognostic indicator for worse OS with increasing T-stage [3,24]. This could be explained by the low number of events (death or progress) in our group compared with the collective of DeAngelis et al. [3,24].

Similar to many reported series in the literature, the hard palate was confirmed as the most common site of ACC of MiSGs, i.e., 53.7%, and most patients were detected in local advanced (T3–4) disease (83.6%) [1,4]. Detection of palatal tumors may be clinically difficult, because clinical symptoms like pain are often notable after perineural invasion. This may lead to diagnostic delay and allow tumor progression with more advanced stages at first presentation [5].

Due to the small number of patients with stage T1 tumors, no statement for adjuvant treatment options in patients with T1, N0 status could be done. This case should always be discussed individually, taking into account the resection margin. Similar to our study, Mücke et al. did not find significant differences between early disease (T1–2) and advanced disease (T3–4), but they observed better survival in patients who were lymph node negative [12].

In the literature, most authors recommend surgery, with the aim of achieving oncological excision margins, followed by adjuvant radiotherapy as standard treatment. Because of its difficult location in the oral cavity, it is not easy to achieve adequate excision margins in this site. The fact that 85.1% patients had a positive surgical margin (R1/2) in our study shows, as in previous studies, the difficulty of achieving adequate excision in this site [4]. In our series, all patients underwent surgery followed by adjuvant bimodal radiotherapy, usually for an incomplete or inadequate excision, but there was no significance. This could be also explained by the low number of events (death or progress) in patients with R0 resection. Even if there were not any significant differences in OS, PFS, and LDFS between different resection margins, disease-free surgical margins are easier to achieve for smaller ACCs (stages T1/2) in the oral cavity than in advanced disease (stages T3/4): Only one patient (1.5%) with negative resection margin (R0) but 10 patients (14.9%) with positive resection margin (R1/2) developed a local failure. This confirms that all patients with positive resection margins have the indication for adjuvant RT.

The toxicity of RT in the oral cavity is crucial for patients’ quality of life [26]; thus, analysis of early and late side effects is of major importance. Acute and late toxicity of bimodal RT is moderate [13]; this statement confirms with our findings. The most common acute toxicities were in 64.2% xerostomia grade 1, 56.7% dermatitis grade 1, and 58.2% dysphagia grade 2. Also, 88.1% of patients suffered moderate fatigue (grade 1). In view of the large volumes treated in this study (median boost volume: 134 mL), this also seems acceptable. Higher grade dysphagia (11.9% grade 3) occurred in patients with bilateral RT of both cervical lymph node regions. Severe late toxicity is rare, and despite complex treatment sites and high applied total doses, only 2 patients reported osteoradionecrosis (ORN) of the upper jaw. ORN, which is characterized by irreversible bone necrosis, is one of the most serious complications of oral RT. Because of recent changes in treatment techniques, the prevalence of ORN ranges from 1% to 56% [27,28,29,30,31]. In recent years, the incidence of ORN has dropped to 10%, but its risk has not been completely eliminated. ORN typically develops more than 2 years after RCT [29]; in our analysis, the follow-up duration was significantly lower for some patients.

The limitations of this study include its retrospective nature, which led to a shortage of necessary data on some single cases. However, we were able to retrieve follow-up data covering a lengthy time period for all patients.

The power of this study is that we were able to show—in a dedicated collective of ACCs of MiSGs in the oral cavity undergoing postoperative radiotherapy with IMRT and carbon ion boost and an extended follow up of 40 months—good control rates with moderate toxicity. OS and PFS were shown to correlate significantly with nodal stage and a positive trend for OS with CTV ≤ 400 ccm).

## 4. Materials and Methods

### 4.1. Demographic and Patient Characteristics

We collected retrospectively Data for 67 patients with ACC of the minor salivary glands who were treated at the Department of Radiation Oncology at the University Hospital of Heidelberg between 2000 and 2018. Minimum follow-up with computer tomography (CT) or magnetic resonance imaging (MRI) and clinical examination was 6 months. We collected basic patient and treatment data from the Heidelberg Nationales Centrum für Tumorerkrankungen (NCT) Cancer Registry. There were 40 female patients (59.7%) and 27 male patients (0.3%). Median patient age was 29 to 83 years (median 58 years). In all cases, the RT was performed after surgical resection. Of the 67 patients, 64 had local advanced disease with T3/4 stage (85.1%), and 16 (23.9%) were lymph node positive. There were 10 patients (14.9%) with negative resection margin (R0), 52 (77.6%) with microscopically (R1) and 5 (7.5%) with macroscopically (R2) resection margin. There was no patient in a metastatic disease at initial diagnoses, as well as at start of RT. All patients were treated with combined RT which includes photons and carbon ions. As part of the initial tumor treatment, RT started within 3 months (range: 1–15 months) after tumor diagnosis and 1.5 months (range: 0.7–13) after surgery. Patient’s main characteristics are listed in Table 3. The study was approved by the ethics committee of the University of Heidelberg, Germany (S-421/2015).

### 4.2. Pre-Treatment Imaging

As part of the radiation planning, all patients underwent a native and contrast-enhanced computed tomography computer tomography (CT) scan at our institution with a slice thickness of 3 mm, and were immobilized with a thermoplastic mask. If there were no contraindications, an additional MRI scan with a contrast agent (gadolinium, T1-weighted, fat-saturated if necessary, slice thickness of 3–5 mm) was performed, and both MRI and CT images were matched to improve radiation planning.

The clinical target volume (CTV) involves microscopic extension of disease and surgical margins; anatomic boundaries were especially respected. Safety margins in form of the planning target volume (PTV) were added with 5–7 mm on the CTV. The radiation dose was prescribed to the PTV (IMRT)/ CTV (C12), and was aimed to be covered by the 90%/95% isodose.

### 4.3. Treatment Modalities — Combined RT (IMRT+C12)

No patient who had an irradiation before, and all underwent a preceding tumor resection. All patients received a mixed beam regimen as described. The additive RT was performed as a combined concept consisting of a combination of photon radiation (3D-planned, image-guided IMRT (TomoTherapy^®^ (Accuray, Sunnyvale, CA, USA)), one fraction per day, 5 fractions per week) and a separate carbon ion boost (3D-planned, image-guided particle therapy, carbon ions, active rasterscanning, alpha/beta = 2, one fraction per day, 6 fractions per week). The total dose was 48–56 Gy photons (single dose 2 Gy) and 18–24 Gy (RBE) carbon ions (single dose 3 Gy (RBE)). The cumulative dose was 68–74 Gy (EQD2). The total dose of 74 GyE corresponds to a biological effective dose of 80 Gy BED. In 60 of 67 patients the cervical lymphatic drainage was included in the clinical target volume (ipsilateral 39 patients (65%), bilateral 21 patients (35%) with a median cumulative total dose of 50 Gy (range 48–66). The main treatment characteristics are listed in Table 4. The median CTV of photon radiation was 346 ccm (range: 21–921 ccm). The median CTV of the carbon ion boost radiation consisted of 134 ccm (range: 21–411 ccm).

### 4.4. Follow-Up

Follow-ups consisted of CT and/or MRI imaging every three months within the first two years after completion of RT, as well as regular clinical examinations to evaluate outcome and potential tumor progression. In years 3–5 after RT, the frequency of CT or MRI imaging and clinical examinations was performed in 6-month intervals. One patient was lost to follow-up. The median follow-up was 40 months (range: 1–200 months) for all patients and 50 months (range: 1–200 months) for living patients.

### 4.5. Overall Survival (OS)

OS was calculated by Kaplan-Meier estimates. The observed time was defined as start of RT until death or the last follow-up. Every known death was counted as event. Patients alive and patients lost to follow-up were counted as censored.

### 4.6. Progression-Free Survival (PFS)

PFS was calculated by Kaplan-Meier estimates. The observed time was defined as start of RT until tumor progression/death or the last follow-up. Locoregional or distant tumor progression were counted as events. Patients without tumor progression and patients lost to follow-up were counted as censored.

### 4.7. Local Disease-Free Survival (LDFS)

LRFS was calculated by Kaplan-Meier estimates. The observed time was defined as start of RT until local tumor progression at the primary tumor site or last follow-up. Patients without local recurrence and patients lost to follow-up were counted as censored.

### 4.8. Treatment Toxicity

Acute toxicity was evaluated at the end of RT. Late toxicity was evaluated 12 weeks after completion of radiation, and was described according to the Common Terminology Criteria for Adverse Events (CTCAE) criteria (version 4.03, U.S. Department of Health and Human Services, Washington, DC, USA).

### 4.9. Statistical Analysis

For statistical analysis, Kaplan-Meier estimates were calculated. These were conducted using IBM SPSS software version 24. The results are presented as mean, range, and percentage. Subgroups were compared using the log-rank test. *p*-values of 0.05 or less were considered statistically significant. Odds ratios were accompanied with 95% confidence intervals. For comparison between groups, the Chi-squared test was performed in categorical and continuous variables.

## 5. Conclusions

The current data present extended follow up data of ACC of MiSGs in oral cavity with good local control rates using combined radiation technique (IMRT plus carbon ions) after radical surgery. There was a significant correlation between positive lymph nodes and inferior survival. The toxicity was low; only two patients developed ORN of the upper jaw, 18 and 66 months after radiotherapy.

## Figures and Tables

**Table 1:** Overview of studies of radiotherapy in patients with adenoid cystic carinomas in our department.

Authors/Year	Number of Patients	Median Follow Up (Months)	RT Modality	Treatment Intention	T4-Stage	LC	OS	Conclusion for Using C12
Jensen et al. 2015 [12]	53	42	Combined (IMRT+C12)	R1, R2, definitive	57%	3-years: 81.9%	3-years: 78.4%	less toxicity in combined group
Jensen et al. 2015 [12]	58	74	Combined (IMRT+C12)	definitive, R2	90%	5-years: 59.6%	10-years: 44.2%	LC, OS, PFS better in combined group
Jensen et al. 2015 [12]	37	63	photons alone	definitive, R2	94%	5-years: 39.9%	10-years: 19.6%	LC, OS, PFS better in combined group
Jensen et al. 2016 [12]	309	34	Combined (IMRT+C12)	R1, R2, definitive	60%	3-years: 83.7%	3-years: 88.9%	good LC in combined group

Abbreviations: overall survival (OS), local control (LC), progression-free survival (PFS), radiotherapy (RT), intensity modulated radiotherapy (IMRT), carbon ions (C12).

**Table 2:** Acute and late treatment toxicity.

Early Treatment Toxicity		No of Patients		Late Treatment Toxicity	No of Patients	
	CTC grade	***n***	%	CTC grade	***n***	%
Mukositis						
	*1*	8	11.9	*1*	7	10.4
	*2*	21	31.3	*2*	2	4.5
	*3*	35	52.2			
Dermatitis						
	*1*	38	56.7	*1*	11	16.4
	*2*	22	32.9			
	*3*	5	7.5			
Dysphagia						
	*1*	13	19.4	*1*	13	19.4
	*2*	39	58.2			
	*3*	8	11.9			
Xerostomia						
	*1*	43	64.2	*1*	33	49.3
	*2*	8	11.9	*2*	7	10.4
Epitheliolysis						
	*3*	11	16.4			
Osteoradionecrosis						
				*3*	2	3.0
Hearing impairment						
		13	19.4		8	11.9
Loss of taste						
		50	74.6			
Trismus						
		16	23.9		20	29.9
Edema						
		2	3.0		1	1.5
Fatigue						
		59	88.1			
Hair loss						
					3	4.5

Abbreviations: common toxicity criteria (CTC).

**Table 3:** Patients’ characteristics.

Characteristics	No of Patients (%)
gender	
male	27 (40.3%)
female	40 (59.7%)
T-stage	
1	6 (9.0%)
2	5 (7.5%)
3	12 (17.9%)
4	43 (64.2%)
N-stage	
0	51 (76.1%)
+	16 (23.9%)
Resection margin	
0	10 (14.9%)
1	52 (77.6%)
2	5 (7.5%)
Locations in oral cavity	
buccal	8 (11.9%)
palate (soft/hard)	35 (52.2%)
tongue	5 (7.5%)
maxilla	19 (28.4%)

Abbreviations: tumor (T), nodes (N), metastases (M).

**Table 4:** Main treatment characteristics.

Characteristics	No of Patients
irradiation	
photons + carbon ions	67
median IMRT dose	**Gy (range)**
	50 (48–56)
median C12 dose	
	24 (18–24)
median dose of cervical lymphatic drainage	
	50 (48–56)
cumulative dose (IMRT + C12)	
	74 (68–74)
median CTV volume (ccm)	
IMRT	346 ccm (range: 21–921 ccm)
C12	134 ccm (range: 21–411 ccm)

Abbreviations: intensity modulated radiotherapy (IMRT), carbon ions (C12), gray (Gy), cubic centimeter (ccm).
